# Type I Interferons and Malaria: A Double-Edge Sword Against a Complex Parasitic Disease

**DOI:** 10.3389/fcimb.2020.594621

**Published:** 2020-12-02

**Authors:** Xiao He, Lu Xia, Keyla C. Tumas, Jian Wu, Xin-Zhuan Su

**Affiliations:** ^1^ Malaria Functional Genomics Section, Laboratory of Malaria and Vector Research, National Institute of Allergy and Infectious Disease, National Institutes of Health, Bethesda, MD, United States; ^2^ Center for Medical Genetics, School of Life Sciences, Central South University, Changsha, China

**Keywords:** IFN-Is, *Plasmodium*, immune response, signaling pathways, virulence

## Abstract

Type I interferons (IFN-Is) are important cytokines playing critical roles in various infections, autoimmune diseases, and cancer. Studies have also shown that IFN-Is exhibit ‘conflicting’ roles in malaria parasite infections. Malaria parasites have a complex life cycle with multiple developing stages in two hosts. Both the liver and blood stages of malaria parasites in a vertebrate host stimulate IFN-I responses. IFN-Is have been shown to inhibit liver and blood stage development, to suppress T cell activation and adaptive immune response, and to promote production of proinflammatory cytokines and chemokines in animal models. Different parasite species or strains trigger distinct IFN-I responses. For example, a *Plasmodium yoelii* strain can stimulate a strong IFN-I response during early infection, whereas its isogenetic strain does not. Host genetic background also greatly influences IFN-I production during malaria infections. Consequently, the effects of IFN-Is on parasitemia and disease symptoms are highly variable depending on the combination of parasite and host species or strains. Toll-like receptor (TLR) 7, TLR9, melanoma differentiation-associated protein 5 (MDA5), and cyclic GMP-AMP synthase (cGAS) coupled with stimulator of interferon genes (STING) are the major receptors for recognizing parasite nucleic acids (RNA/DNA) to trigger IFN-I responses. IFN-I levels *in vivo* are tightly regulated, and various novel molecules have been identified to regulate IFN-I responses during malaria infections. Here we review the major findings and progress in ligand recognition, signaling pathways, functions, and regulation of IFN-I responses during malaria infections.

## Introduction

**Figure 1:**
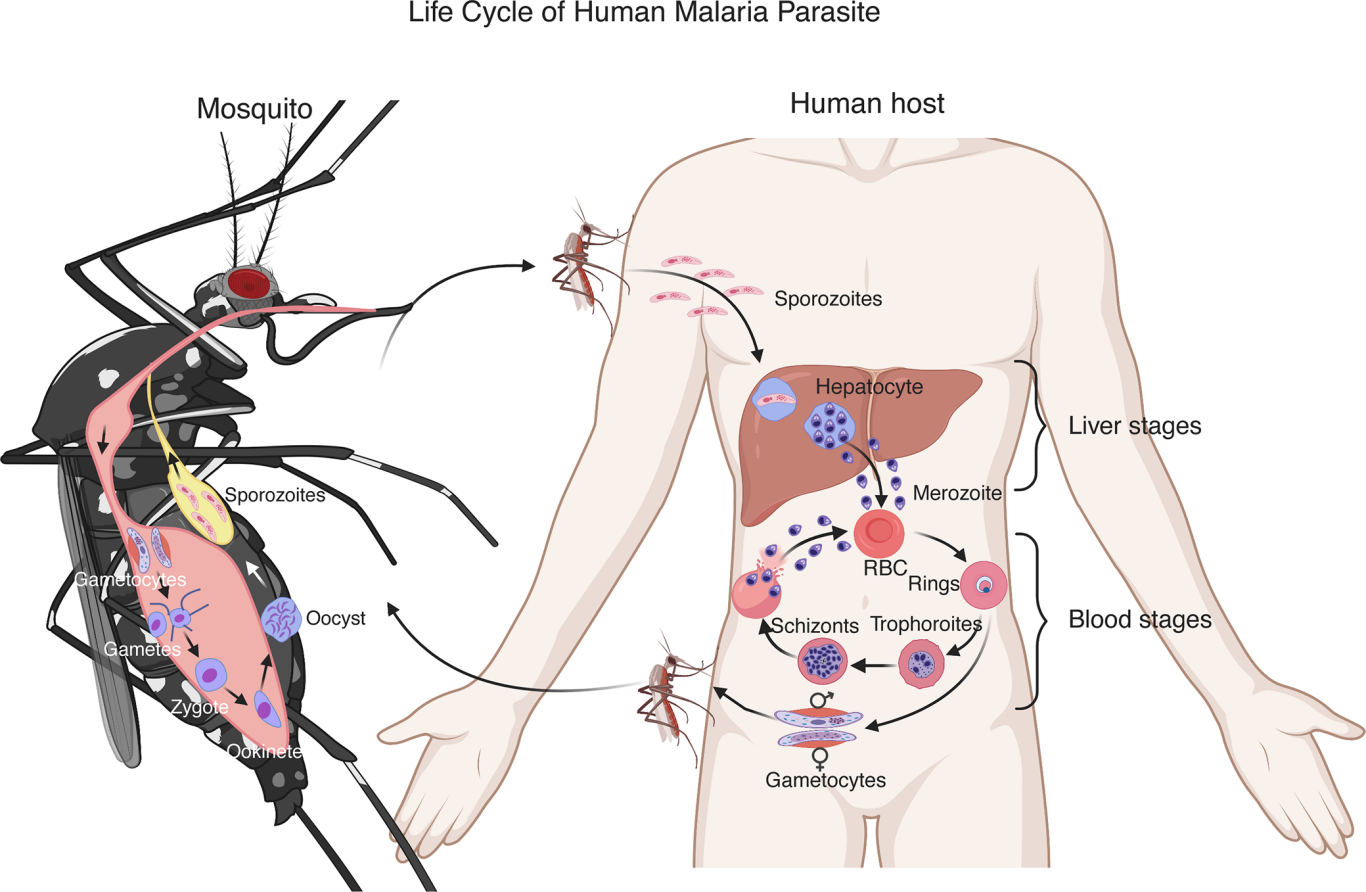
Life cycle of a human malaria parasite. Malarial infection begins when an infected female *Anopheles* mosquito bites a human host and injects sporozoites into the skin. The sporozoites then travel through the bloodstream to invade hepatocytes. A sporozoite multiplies in the hepatocyte to generate thousands of merozoites. After being released from the liver, the merozoites invade red blood cells (RBCs) and develop to ring stage, trophozoite and schizont that contains 16–32 merozoites. The merozoites are released after rupture of infected RBCs to invade new erythrocytes. These blood stages of the parasite life cycle cause most malaria symptoms. A small number of merozoites develop into sexual stages, male and female gametocytes. Gametocytes are taken up by a second mosquito through a blood meal. In the mosquito midgut, the gametocytes differentiate into gametes, zygotes, ookinetes and then oocysts. A mature oocyst contains tens of thousands of sporozoites that migrate to the mosquito salivary glands to be injected into another human host to start a new cycle.

*Plasmodium* parasites have a complex life cycle developing within a vertebrate host and a female *Anopheles* mosquito (**Figure 1**). Malaria infection begins with a mosquito bite injecting sporozoites into the skin of a vertebrate host. The sporozoites travel to the liver through the bloodstream and infect hepatocytes. In the liver, the parasites undergo multiple rounds of replication, resulting in thousands of merozoites (Prudencio et al., 2006). The merozoites are released into the blood where they rapidly infect red blood cells (RBCs) and begin intraerythrocytic cycle of replication, releasing more merozoites to invade new RBCs. The erythrocytic development cycle takes approximately 24 h for *Plasmodium knowlesi* and rodent parasites such as *Plasmodium yoelii*, *Plasmodium berghei*, and *Plasmodium chabaudi*, 48 h for *Plasmodium falciparum*, *Plasmodium vivax*, and *Plasmodium ovale*, and 72 h for *Plasmodium malariae*. The asexual intraerythrocytic cycle is responsible for malaria symptoms (Crompton et al., 2014). During the development in RBCs, the parasites express various proteins on infected RBCs (iRBCs) and release a large amount of different materials into the bloodstream when iRBCs rupture, which trigger vigorous host immune responses and malaria signs or symptoms, including fever and chills, headache, anemia and possibly cerebral malaria.

**Figure 2:**
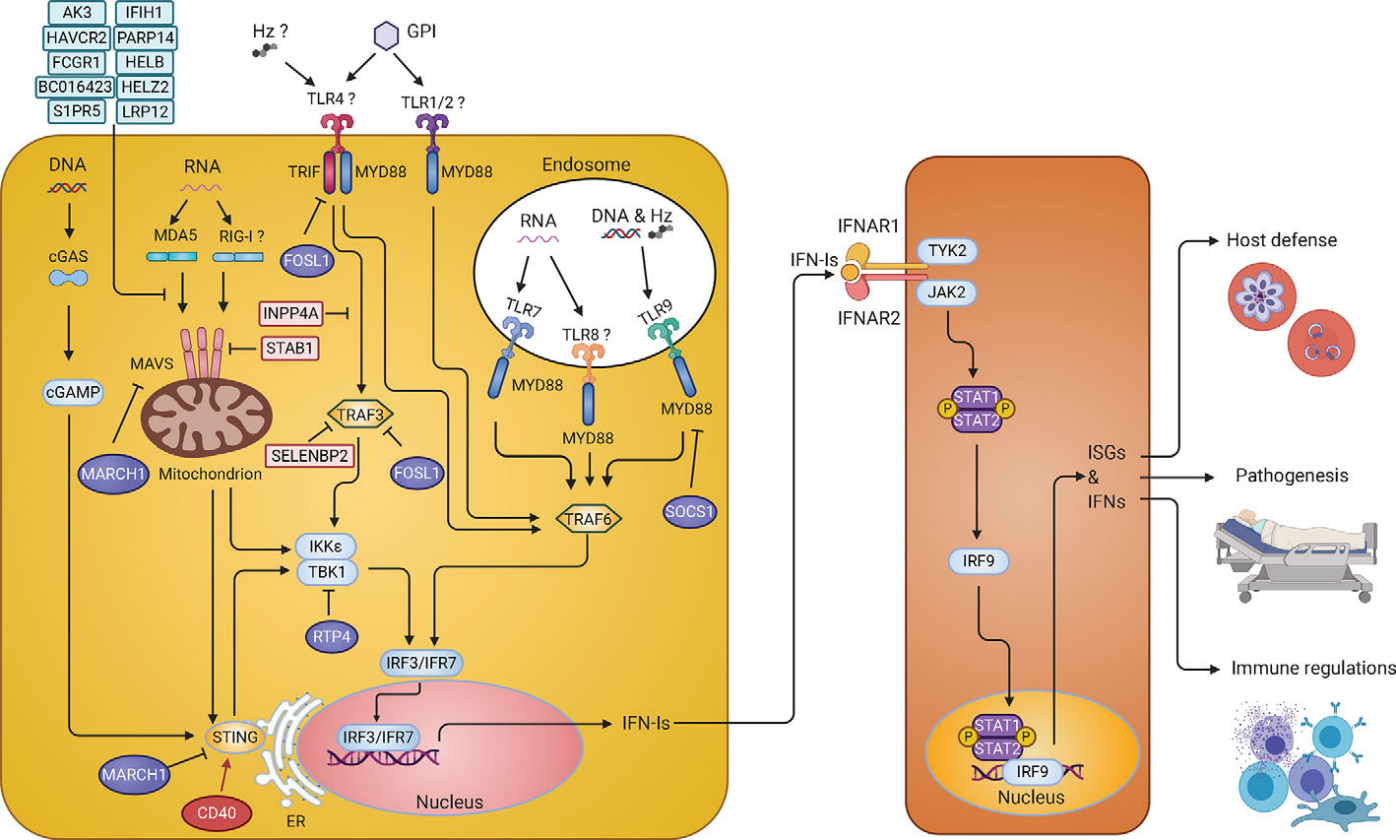
IFN-I response and signaling pathways in malaria parasite infections. Parasite pathogen-associated molecular patterns (PAMPs) such as DNA and RNA are recognized by Toll-like receptors (TLRs) on the membrane of endosomes, including TLR7, TLR9, and possibly TLR8, and activate MYD88-TRAF6-IRF7 signaling cascade to stimulate production of IFN-Is. In the cytosol, parasite RNA is sensed by MDA5 (maybe RIG-I too) leading to the activation of MAVS-TBK1-IRF3 signaling and IFN-I production. Parasite DNA in the cytosol is sensed by cGAS, resulting in the activation of STING-TBK1-IRF3 signaling pathway to produce IFN-Is. On the cell surface, glycosylphosphatidylinositol (GPI) might be recognized by TLR4 and/or TLR1/2 that activates MYD88-TRAF6-IRF7 and TRIF-TBK1-IRF3 pathways to produce IFN-Is. Hemozoin (Hz) can form a complex with DNA and be recognized by TLR9 or with host fibrinogen and recognized by TLR4. Hz itself can also be sensed by NLR family pyrin domain containing 3 (NLRP3). However, recognition of Hz by these receptors mostly leads to the production of the proinflammatory cytokines. Many molecules (in light blue or light pink) are regulators that have been shown to inhibit IFN-I responses *in vitro*. The roles of CD40, FOSL1, MARCH1, and RTP4 in regulating IFN-I responses were further demonstrated *in vivo* during malaria parasite and/or viral (RTP4) infections. These regulators either can increase (CD40 in red) or decrease (FOSL1, MARCH1, and RTP4 in purple) IFN-I production through phosphorylation and/or ubiquitination of proteins in the IFN-I response pathways. The IFN-Is secreted by the cells bind to the IFNAR to activate the STAT1/STAT2-IRF9 pathway leading to transcription of ISGs that either directly act on parasites or modulate host immune responses. Infection of *Ifnar1^-/^* ^-^ mice will affect downstream IFN-I signaling but may not influence (and possibly stimulate) IFN-Is production.

Innate immunity is the first line of host defense against an invading pathogen. Interferons (IFNs) are produced by the host immune system and are well recognized for their role in antiviral infections. IFNs were initially described in 1957 as soluble glycoproteins with strong effects to “interfere” with the virus replication (Isaacs and Lindenmann, 1957; Isaacs et al., 1957). Today, three major groups of IFNs have been characterized: Type I IFN (IFN-I), IFN-II and IFN-III (Borden et al., 2007; Kotenko, 2011). IFN-Is consist of 13 IFN-α subtypes in humans (14 in mice), IFN-β, IFN-ω, IFN-κ, IFN-ϵ, IFN-ζ, IFN-δ, and IFN-τ (Pestka et al., 2004; Borden et al., 2007). Among them, IFN-α and IFN-β are the most abundant and well-studied. All IFN-I subtypes signal in an autocrine and paracrine fashion through heterodimeric IFN-I receptor (IFNAR) composed of two subunits, IFNAR1 (IFN-α/β receptor α chain) and IFNAR2 (IFN-α/β receptor β chain) (Uze et al., 2007; Boxx and Cheng, 2016). Binding of IFN-Is to IFNAR induces a cascade of downstream signaling events to initiate the transcription of hundreds of interferon-stimulated genes (ISGs) (**Figure 2**). ISGs include antimicrobial proteins, chemokines/cytokines and inflammation-inducing mediators. Many ISGs target critical molecules and pathways of a pathogen directly. Chemokines/cytokines and their receptors enable cell-to-cell communication and cell migration, whereas negative regulators of signaling pathways maintain a balanced IFN response and cellular homeostasis. Other ISGs encode for proapoptotic proteins, leading to cell death under certain conditions (Schneider et al., 2014; Lukhele et al., 2019).

IFN-Is are some of the most common cytokines produced by the host and play important roles in both protection and pathogenesis during malaria parasite infections (Angulo and Fresno, 2002; Stevenson and Riley, 2004; Schofield and Grau, 2005; Clark et al., 2008; Dunst et al., 2017; Gotz et al., 2017). At the early stage of infection, the parasites can be recognized by the host, which induces complex immune responses, including production of IFNs and pro-inflammatory cytokines (Scragg et al., 1999). The pathogen components recognized by host pathogen recognition receptors (PRRs) are called pathogen-associated molecular patterns (PAMPs). PAMPs are generally evolutionarily conserved molecules, including DNA, RNA, lipopolysaccharide (LPS), peptidoglycans, glycosylphosphatidylinositol (GPI), and some proteins (Beutler, 2009; Takeuchi and Akira, 2010; Kawasaki and Kawai, 2014; Wu and Chen, 2014; Brubaker et al., 2015; Vijay, 2018). During infections, some host molecules that are normally not accessible to the host immune system can be ‘released’ and trigger immune responses. These molecules such as high mobility box 1 (HMGB1), heat shock proteins (HSPs), SP100 protein family, and uric acid crystal are called danger-associated molecular patterns (DAMPs) (Rubartelli and Lotze, 2007; Tang et al., 2012; Kaczmarek et al., 2013; Venereau et al., 2015). The host can detect PAMPs and DAMPs by a wide range of PRRs, leading to activation of downstream pathways and production of chemokines/cytokines. PRRs are present at many different cellular locations, including the outer plasma membrane, endosomal membrane luminal surface, mitochondria outer membrane and cytosol (Kawai and Akira, 2011; Kawasaki and Kawai, 2014; Wu and Chen, 2014). Toll-like receptors (TLRs), RIG-I-like receptors (RLRs), cyclic GMP-AMP synthase (cGAS), nucleotide-binding oligomerization domain (NOD)-like receptors (NLRs), and absent in melanoma 2 (AIM2) are common receptors that recognize PAMPs and host DAMPs to initiate IFN-I and inflammatory responses.

Initially identified as potent antiviral mediators, IFN-Is have been shown to play important roles against many pathogens, including bacteria, fungi, and protozoa (Ramirez-Ortiz et al., 2011; Beiting, 2014; Boxx and Cheng, 2016; Silva-Barrios and Stager, 2017). However, IFN-Is can also cause immunopathology in acute viral infections (Davidson et al., 2014) and lead to immunosuppression during chronic viral infections (Teijaro et al., 2013; Wilson et al., 2013). Similarly, in some bacterial infections, IFN-Is have been shown to exacerbate infections and promote secondary or chronic bacterial infections (Boxx and Cheng, 2016). Dual and opposing roles of IFN-Is have also been reported in many protozoan infections (Beiting, 2014; Silva-Barrios and Stager, 2017), including conflicting roles in malaria infections. Here we review and discuss IFN-I responses during malaria infections, including parasite ligands, host receptors, IFN-I induction and regulation, and their functions in disease pathology and protection.

## Sensing Malaria Parasites: Ligands and Receptors

Many PRRs have been reported to recognize different malaria PAMPs or DAMPs to initiate the immune responses (**Figure 2**). TLRs, RLRs and cGAS have been shown to play important roles in IFN-I responses during malaria parasite infections, whereas NLRs and AIM2 largely contribute to activation of inflammasomes (Gazzinelli et al., 2014). Malaria parasite hemozoin (Hz), RNA, DNA, GPI as well as host derived uric acid crystal, peroxiredoxins and extracellular vesicles (EVs) are examples of PAMPs and DAMPs that can induce IFN-I and inflammatory responses. TLRs consist of 10 members in humans (TLR1–TLR10) and 12 receptors in mice (TLR1–TLR9 and TLR11–TLR13) (Takeda et al., 2003; Nie et al., 2018). The RLRs are located at the cytoplasm and contain a DexD/H box RNA helicases domain that can detect RNA (Loo and Gale, 2011). There are three known RLRs: retinoic acid-inducible gene I (RIG-I), melanoma differentiation-associated protein 5 (MDA5), and DExH-box helicase 58 (DHX58 or LGP2). RIG-I and MDA5 are responsible for sensing double-stranded RNA (dsRNA), whereas DHX58 functions as a regulator of the RIG-I and MDA5 signaling pathways (Yoneyama et al., 2005). cGAS binds to microbial DNA and self-DNA in the cytoplasm and catalyzes the synthesis of cyclic guanosine monophosphate–adenosine monophosphate (cGAMP) (Sun et al., 2013). cGAMP acts as a second messenger to activate the downstream signal adaptor, stimulator of interferon genes (STING) (Wu et al., 2013).

TLR1, TLR2, TLR4, TLR6, TLR7 and TLR9 have been reported to recognize malaria ligands. TLR2 forms heterodimers with TLR1 or TLR6, and the heterodimers can bind to the distinct structure of parasite GPI (Campos et al., 2001; Krishnegowda et al., 2005). TLR4 can recognize parasite GPI as well as the peroxiredoxins and fibrinogen bound to Hz (Krishnegowda et al., 2005; Zhu et al., 2005; Barrera et al., 2011). TLR7 and TLR8 sense parasite RNA (Baccarella et al., 2013; Spaulding et al., 2016; Yu et al., 2016; Coch et al., 2019), and TLR9 recognizes parasite DNA and Hz-DNA complexes (Pichyangkul et al., 2004; Parroche et al., 2007; Wu et al., 2010; Gowda et al., 2011). The major cytosolic receptor for sensing malaria RNA is MDA5 (Liehl et al., 2014; Miller et al., 2014), although RIG-I has also been implicated to play a role in response to malaria infection (Wu et al., 2020). cGAS recognizes parasite DNA and leads to the production of IFN-Is (Yu et al., 2016; Gallego-Marin et al., 2018; Hahn et al., 2018). It is possible that different sensors are activated by parasite products at different developmental stages, leading to variation in levels of IFN-I subtypes over time. Additionally, different IFN-I subtypes can have different biological and immunological roles. For example, C57BL/6J mice infected with lymphocytic choriomeningitis virus (LCMV) Cl-13 strain produced both IFN-α and IFN-β. However, IFN-α was found to control early viral dissemination, whereas IFN-β could suppress T cell responses and delay clearance of persistent virus (Ng et al., 2015; Ng et al., 2016). Additionally, IFN-α and IFN-β were shown to have different activities against West Nile virus infection (Sheehan et al., 2015). Whether IFN-α and IFN-β play different roles in malaria parasite infections requires further investigations.

### DNA Sensing and Signaling Pathways

Many studies have shown that endosomal TLR9 and cytoplasmic cGAS can recognize microbial DNA and host self-DNA in certain pathological conditions (**Figure 2**) (Pichyangkul et al., 2004; Beutler, 2009; Takeuchi and Akira, 2010). During malaria infections, TLR9 binds unmethylated CpG motifs of parasite DNA (Lamphier et al., 2006; Ohto and Shimizu, 2016). It is worth noting that *P. vivax* parasite that has a higher frequency of CpG motifs in the genome than *P. falciparum* parasite is also a stronger fever inducer (Anstey et al., 2012). Malaria parasites in the iRBCs or free merozoites are phagocytized by dendritic cells (DCs) and macrophages and enter the endosomes of these cells (Parroche et al., 2007; Wu et al., 2010; Gowda et al., 2011; Hirako et al., 2015). The endosomes then fuse to the lysosomes to form phagolysosomes and release DNA into the acidic environment. There, DNA can bind to TLR9 and activate the mitogen-activated protein kinase (MAPK)/nuclear factor kappa-light-chain-enhancer of activated B cells (NF-κB)/interferon regulatory transcription factor (IRF) 7 pathways, leading to the production of IFN-Is and proinflammatory chemokines/cytokines (Sasai et al., 2010; Kawai and Akira, 2011). For the IRF7 pathway, TLR9 recruits the toll/interleukin-1 receptor (TIR) domain containing adaptor protein (TIRAP) that binds to myeloid differentiation primary response 88 (MYD88) and interleukin-1 receptor-associated kinase 1 (IRAK1) (Kawagoe et al., 2008; Barton and Kagan, 2009; Kawai and Akira, 2010). The MYD88-IRAK1 complex further recruits tumor necrosis factor (TNF) receptor associated factor (TRAF) 3 and IRF7 that translocate into the nucleus to activate promoters of IFN-I genes (O’Neill et al., 2003; Kawai and Akira, 2008).

Parasite DNA can also enter the cytosol and activate cGAS and AIM2. cGAS synthesizes cGAMP that binds to STING (also known as MITA, ERIS or MPYS) (Ishikawa and Barber, 2008; Zhong et al., 2008; Sun et al., 2009; Jin et al., 2013) and triggers conformational changes of STING to form STING oligomers through side-by-side packing of the STING molecules (Shang et al., 2012). STING then translocates from the endoplasmic reticulum (ER) to the Golgi apparatus (Gui et al., 2019) to bind TANK binding kinase 1 (TBK1), leading to phosphorylation of interferon regulatory factor (IRF) 3 (Hornung and Latz, 2010). Phosphorylated IRF3 also translocates into the nucleus to activate IFN-I genes. DNA sensing by AIM2 generally triggers the production of inflammatory cytokines and activation of inflammasomes (Kalantari et al., 2014). Therefore, TLR9 and cGAS/STING are the major sensors for recognizing malaria parasite DNA for IFN-I responses.

### RNA Sensing and Signaling Pathways

Parasite RNA from both liver and blood stages can be recognized by the host RNA sensors (**Figure 2**). During development in the liver, parasite RNA is mainly recognized by MDA5 (Liehl et al., 2014; Miller et al., 2014). MDA5 becomes activated after binding RNA and releases its caspase activation and recruitment domains (CARD) from the C-terminal regulatory domain (Cui et al., 2008). Activated MDA5 recruits mitochondrial antiviral-signaling protein (MAVS) through homotypic CARD-CARD domain interactions (Kawai et al., 2005; Meylan et al., 2005; Seth et al., 2005; Xu et al., 2005). MAVS forms prion-like structures and further recruit TRAF3, TBK1 and IκB kinase epsilon (IKKϵ) to phosphorylate IRF3 and IRF7, leading to IFN-I production (Hou et al., 2011; Jacobs and Coyne, 2013). MAVS can also bind to TRAF6 and activate NF-κB/IKK complex, resulting in the production of inflammatory cytokines (Belgnaoui et al., 2011). TLR7 and MDA5 can also sense parasite RNA from blood stages (Baccarella et al., 2013; Wu et al., 2014; Spaulding et al., 2016; Yu et al., 2016). Similar to TRL9, TLR7 activates the MYD88 and IRF7 pathway to produce IFN-Is (Baccarella et al., 2013; Yu et al., 2016). TLR7 and MDA5 are the major sensors for malaria parasite RNA for IFN-I responses.

Additionally, RNA polymerase III (pol III) was reported to convert AT rich viral DNA into dsRNA that can be recognized by the RLRs (Chiu et al., 2009). Pol III deficiency led to reduced IFN-I production in Raw 264.7, but not in HEK293T cells (Sharma et al., 2011; Wu et al., 2014). This discrepancy is likely due to difference in gene expression between the two cell lines including the lack of TLR expression in the HEK293T cells (Hornung et al., 2002). Interestingly, RIG-I expression was detected in a study using trans-species expression quantitative trait locus (ts-eQTL) analysis after infection of 24 parasite progeny from a *P. y. yoelii* 17XNL × *P. y. nigeriensis* N67 cross (Wu et al., 2020). Additionally, messenger RNA (mRNA) and protein levels of IFN-β could be significantly suppressed by RNAi knockdown of the genes encoding MDA5, RIG-I, or RNA pol III after *P. yoelii* DNA stimulation (Wu et al., 2014). However, whether RIG-I and Pol III play a role in the recognition of parasite DNA or RNA *in vivo* requires further investigations.

### Responses to GPI

GPI exists in many eukaryote species and is attached to a protein during posttranslational modification (Brown and Waneck, 1992). The major function of GPI is binding to certain functional proteins at the cell surfaces (Ferguson et al., 1999). GPI contains a conserved ethanolamine phosphate-substituted oligosaccharide moiety that links to phosphatidylinositol with an alpha glycosidic bond (Ropert and Gazzinelli, 2000). The structures of GPIs in different species vary, mainly in the lipid substituents, sugar chain length, or ethanolamine phosphate residues attached to the conserved glycan moiety. Malaria GPI is one of the earliest identified parasite PAMPs (Schofield and Hackett, 1993). The detailed structures of parasite GPI have been studied using biochemical analysis and mass spectrometry (Naik et al., 2000). The structures of parasite GPIs are different from human GPIs and can be recognized by the host’s immune system. Studies have shown that malaria GPI is sensed by TLRs on the surface of macrophages or DCs, mainly through the TLR1-TLR2 heterodimers and TLR4 (Krishnegowda et al., 2005; Zhu et al., 2005; Lu et al., 2006; Zhu et al., 2009). Similar to TLR7 and TLR9, TLR1-TLR2 and TLR4 recruit MYD88 to activate the NF-κB and IRF7 pathways, leading to the production of IFN-Is and inflammatory cytokines. TLR4 can also recruit a protein called TIR-domain-containing adapter-inducing interferon-β (TRIF) that further forms a complex with IKKϵ and TBK1 to phosphorylate and activate IRF3 and IRF7 (Sweeney et al., 2004; Kawagoe et al., 2008; Beutler, 2009; Ning et al., 2011; Kawasaki and Kawai, 2014). However, the majority of studies on malaria GPI have focused on activation of inflammatory cytokines. One study shows that GPI can induce interferon-sensitive response element (ISRE) activation *in vitro*, which suggests that GPI may also stimulate IFN-I production *via* TLR4 (Yao et al., 2016). Since inflammatory responses by malaria infection can inhibit MYD88-IRF7-dependent IFN-I signaling (Yu et al., 2018), it is possible that the strong pro-inflammatory responses stimulated by parasite GPI can influence other IFN-I pathways indirectly.

### Parasite Hz and Host Responses

Hz is a brown pigment that is formed during the digestion of hemoglobin in the digestive vacuole (DV) of blood stage parasites (Pandey and Tekwani, 1996; Arese and Schwarzer, 1997; Pagola et al., 2000; Jani et al., 2008; Olivier et al., 2014). Hz is captured by immune cells after rupture of iRBCs (Celada et al., 1983; Ho and Webster, 1989; Arese and Schwarzer, 1997). There is no known receptor for Hz; however, it can trigger immune responses in multiple ways. Hz carrying parasite DNA is a potent activator of TLR9, leading to production of ISGs and pro-inflammatory cytokines (Parroche et al., 2007). The parasite DNA does not colocalize with Hz that is synthesized in the DV (Jaramillo et al., 2009). Therefore, Hz probably binds DNA released from dead cells or DV. Hz can also form a complex with the host fibrinogen that is then recognized by TLR4 or CD11b/CD18-integrin on monocytes, leading to production TNF and reactive oxygen species (ROS) (Barrera et al., 2011).

Hz may cause immune cell dysfunction or apoptosis after phagocytosis by the monocytes or macrophages. Studies have shown that macrophages became functionally impaired after ingestion of *P. falciparum*-infected erythrocytes or isolated Hz due to a long-lasting oxidative burst (Schwarzer et al., 1992). Monocytes or macrophages ingesting a large amount of iRBC or Hz may undergo apoptotic death with low levels of cytokine production due to phagolysosomal acidification (Schwarzer et al., 2001; Wu et al., 2015b). Hz can also inhibit differentiation and maturation of human DC by impairing expression of major histocompatibility complex class II antigen (Schwarzer et al., 1998; Skorokhod et al., 2004). Hz induces phagolysosomal destabilization leading to release of lysosomal contents to the cytosol, including DNA or RNA to induce IFN-I responses. Additionally, Hz itself can activate the NLRP3 inflammasome through Lyn and Syk signaling pathways (Dostert et al., 2009; Olivier et al., 2014). An unidentified ligand released from the parasite lysosome (or DV) has been reported to activate NLRP12 inflammasome (Ataide et al., 2014). Overall, Hz is considered as a danger signal which can activate diverse pathways to influence IFN-I production and inflammatory responses (Dostert et al., 2009; Shio et al., 2009).

### Extracellular Vesicles (EVs) and Host Responses

EVs are lipid bilayer-delimited particles derived from iRBCs or other host cells (Babatunde et al., 2020). Based on their size and biological functions, EVs are classified into two forms: exosomes and microvesicles. Exosomes are smaller vesicles ranging from 30 to 150 nm in diameter and are usually released by reticulocytes during differentiation and maturation (Johnstone et al., 1987). Microvesicles are larger vesicles with sizes of 150 nm to 1–2 μm in diameter and are produced by the plasma membrane budding and fission (Tricarico et al., 2017). EVs contain lipids, proteins, and nucleic acids (DNA and RNA) (Laulagnier et al., 2004; Nolte-’t Hoen et al., 2012; Tauro et al., 2012; Ridder et al., 2014; Sinha et al., 2014; Williams et al., 2014; Babatunde et al., 2018). Malaria parasite infection causes a strong inflammatory response, and inflammatory cytokines such as TNF-α can greatly contribute to the production of the EVs by almost all cell types (Combes et al., 1999; Freyssinet, 2003). Studies have shown that EVs containing parasite materials are potential triggers of proinflammatory innate immune responses (Couper et al., 2010). In animal models, blockage of EV production in mice resulted in complete resistance to cerebral malaria development (Combes et al., 2005; Penet et al., 2008). Transfer of EVs from *P. berghei* ANKA-infected mice caused damages to the blood-brain barrier in the recipient mice (El-Assaad et al., 2014). Parasite DNA in the EVs can be detected by cGAS to activate the STING-dependent pathway leading to the production of IFN-Is and inflammatory cytokines (Sisquella et al., 2017). Parasite RNA in the EVs, on the other hand, will activate MDA5 and trigger the MAVS-dependent pathway to produce IFN-Is (Ye et al., 2018). EVs also contain microRNA (miRNA) and mRNA. mRNA can be translated into protein in the recipient cells; meanwhile, miRNA may regulate gene expression in the target cells (Ratajczak et al., 2006; Valadi et al., 2007). Similar to Hz, EVs and their contents can influence several host pathways, including IFN-I responses.

## Host Cells Producing IFN-Is

IFN-Is can be produced by several types of host cells during malaria infections. *Plasmodium* parasites reside in different cell types during their life cycle. In the liver, the parasites develop mostly in the hepatocytes, and large amount of parasite materials are synthesized and released within the hepatocytes. MDA5 in hepatocytes can recognize parasite RNA from liver stages leading to IFN-I production (Liehl et al., 2014; Miller et al., 2014). In the blood stage infections, IFN-I production is more complicated. The parasites reside in RBCs that lack PRRs and the machinery for IFN-I responses. Peripheral blood mononuclear cell (PBMC) cultures stimulated with iRBC can produce IFN-α *in vitro* (Montes de Oca et al., 2016). Other studies find that plasmacytoid dendritic cells (pDC) are the major source of IFN-Is (deWalick et al., 2007; Voisine et al., 2010). Splenic conventional dendritic cells (cDC) are an alternative source of IFN-Is (Voisine et al., 2010; Haque et al., 2014). Macrophages can also produce IFN-Is during blood stage infections (Spaulding et al., 2016; Yu et al., 2016). NK cells from healthy donors’ PBMCs co-cultured with *P. falciparum* 3D7-infected erythrocytes upreguated IFN-α related genes (Grangeiro de Carvalho et al., 2011). Therefore, hepatocytes and DCs are the main sources of IFN-Is during liver and blood stage infections, although many other cell types may also contribute to the IFN-I production.

## IFN-I Responses to Liver Stages

**Figure 3:**
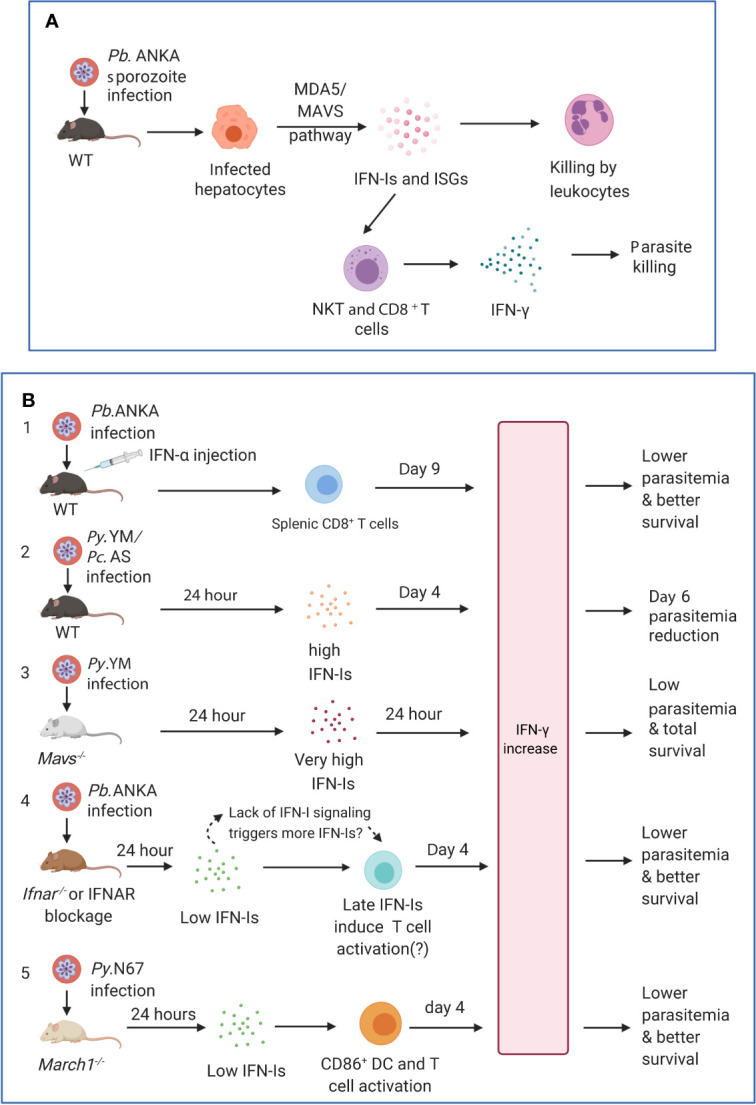
Timing and levels of interferon production may determine the outcomes of malaria parasite infections. **(A)** The protection against liver stages relies on the activation of innate immunity involving IFN-I responses and immune cell infiltration. Infection of C57BL/6 mice with *P. berghei* ANKA liver-stages induces an IFN-I response through MDA5 signaling pathway in hepatocytes. Infiltrating leukocytes (macrophages and neutrophils) are mobilized to the vicinity of infected hepatocytes by IFN-I signaling (Liehl et al., 2014). IFN-γ–secreting immune cells, in particular CD1d-restricted NKT cells, are also likely the main players responsible for the innate elimination during liver stage (Miller et al., 2014). **(B)** Several models show that IFN-Is likely function through regulating IFN-γ production, T cell activation, and adaptive immune response to influence parasitemia and disease severity during blood stage infections. First, several studies showed that injections of IFN-α and/or IFN-β were protective (**Supplementary Table 1**). C57BL/6 mice infected with *P. berghei* ANKA and injected with IFN-α had significantly (*P <*0.05) higher levels of IFN-γ (772 ± 73 pg/ml, day 9 pi) than those receiving diluent (180 ± 14 pg/ml), reduced parasitemia, and better host survival (Vigario et al., 2007). Second, C57BL/6 mice infected with *P. y. nigeriensis* N67 or *P. chabaudi* AS had elevated levels of IFN-α (~320 and ~450 pg/ml, respectively) 24 h pi (Kim et al., 2012; Wu et al., 2020). *Mavs^−/−^* mice infected with *P. y. nigeriensis* N67 or *Ifnar^−/−^* mice infected *P. chabaudi* AS had increased day 6 parasitemia (parasitemia increases in *Ifnar^−/−^* mice may not be significant but had reduced ability to resolve parasitemia later) (Voisine et al., 2010; Kim et al., 2012; Wu et al., 2014). IFN-Is appear to work with IFN-γ in controlling parasitemia during early infection. Compared with *P. y. yoelii* YM infected WT C57BL/6 mice that produced very low IFN-Is 24 h pi, *P. y. nigeriensis* N67-infected mice had significantly higher IFN-α/β, IFN-γ and IL-6 24 h pi (Wu et al., 2020). Additionally, *Ifnar1^−/−^Ifngr1^−/−^* mice infected with *P*. *chabaudi* AS exhibited higher mortality than WT or *Ifnar1^−/−^* mice and were not able to completely clear parasites (Kim et al., 2012). Third, *Mavs^−/−^* mice infected with *P. y. yoelii* YM produced very high levels of IFN-α (~2,800 pg/ml), IFN-β (~2,000 pg/ml) and IFN-γ (>1,200 pg/ml) 24 h pi and all survived the infections (Yu et al., 2016). All these models suggested early production (24 h) of IFN-α/β and IFN-γ can help control parasitemia and may improve host survival. Fourth, in *P. berghei* ANKA-infected WT C57BL/6 mice, low levels of IFN-α/β were observed 24 h after pi (Haque et al., 2011; Haque et al., 2014). Depletion or blockage of IFN-I signaling using *Ifnar^−/−^* mice or anti-IFNAR antibody treatment results in higher levels of IFN-γ, better parasite control, and improved host survival (Haque et al., 2011; Haque et al., 2014). Interestingly, higher levels of IFN-α were observed in WT mice 48 h pi (~70 pg/ml IFN-α vs ~20 pg/ml at 24 h) and day 4 pi (~200 mg/ml IFN-α), suggesting that blockage of IFN-I signaling may stimulate IFN-I response. Unfortunately, the IFN-I levels in *Ifnar^−/−^* mice or mice treated with anti-IFNAR were not measured at additional time points. Similarly, anti-IFNAR antibody treatment of C57BL/6 mice infected with *P. y. yoelii* 17XNL significantly reduced days 16 and 21 parasitemia through inhibition of T regulatory 1 response, enhancement of Tfh cell accumulation and better humoral immunity (Zander et al., 2016). Again, the levels of IFN-Is in the anti-IFNAR treated animals were not reported. Serum levels of IFN-γ between anti-IFNAR antibody treated and non-treated mice were similar at day 16 pi when parasitemia began to show significant difference. It is possible that the lack of IFN-I signaling in these models prompts the system to produce more IFN-Is, and that IFN-Is and IFN-γ work together through regulating immune cell populations and antibody production to control the infections. Fifth, *March1^−/−^* mice infected with *P. y. nigeriensis* N67 (or *P. y. yoelii* YM) had low levels of IFN-Is 24 h pi but had significantly increased IFN-γ and IL-10 day 4 pi due to decreased degradation of CD86/MHCII and T cell activation, leading to reduced parasitemia and better host survival (Wu et al., 2020). IFN-γ was shown to be a key player in controlling parasitemia and host survival. These observations suggest key roles of early IFN-Is (24 h pi) and IFN-γ in later stages of infection (day 4 or later) and emphasize the importance of measuring IFN-Is, IFN-γ, and other cytokines during the course of blood infection for better understanding of protection mechanisms mediated by IFNs. *Pb*.ANKA, *P. berghei* ANKA; *Py*.N67, *P. y. nigeriensis* N67; *Py*.YM, *P. y. yoelii* YM; *Pc.*AS, *P. chabaudi* AS.

Although infection at the liver stages is generally asymptomatic, studies have shown that a complex series of immune events occur during liver stage development, including IFN-I responses (Liehl and Mota, 2012; Liehl et al., 2014; Miller et al., 2014). Transcriptomic analysis of liver stages showed an IFNAR1-dependent IFN-I response in hepatocytes, which was not restricted to *Plasmodium* species (*P. berghei* and *P. yoelii*) nor mouse host strains (BALB/c and C57BL/6) (Liehl et al., 2014). *Plasmodium* parasite RNA was reported to be recognized by MDA5 in hepatocytes (and possibly other receptors). Furthermore, the magnitude of IFN-I responses was parasite dose and replication dependent. Leukocytes were mobilized as effectors by IFN-Is to eliminate the parasites (Liehl et al., 2014) (**Figure 3A**). IFN-I signaling helps recruit NK cells and NKT cells, especially IFN-γ secreting CD1d-restricted NKT cells to eliminate the liver stage parasites (Miller et al., 2014). The IFN-I responses also benefit the host from an immediate liver stage reinfection by significantly reducing the liver parasite load as well as blood stage parasitemia (Liehl et al., 2015). Similarly, attenuated *P. yoelii* parasites that only develop to late liver-stage forms induce significant IFN responses (including IFN-Is and IFN-γ) and provide cross protection against a secondary liver stage infection (*P. yoelii* or *P. berghei*) (Miller et al., 2014). A distinct population of CD11c^+^ cells was also observed to invade the liver at 36–40 h post *P. y. yoelii* 17XNL sporozoite inoculation (Kurup et al., 2019a). These monocyte derived CD11c^+^ dendritic cells (DCs) showed strong IFN-I profiles, with highly upregulated expression of key molecules such as TLR7 and IRF7 in the IFN-I pathways. The CD11c^+^ DCs acquired and presented parasite antigen by phagocytosing infected hepatocytes, to prime CD8^+^ T cells, the major mediators in liver stage protection (Kurup et al., 2019a). *P. berghei* radiation-attenuated sporozoites (RAS) could also induce liver CD8α^+^ DCs that activated CD8^+^ T cells in the liver (Jobe et al., 2009), although the activation level was much weaker than that induced by normal sporozoites (Parmar et al., 2018). The accumulation and activation of CD8α^+^ DCs was associated with higher-levels s of CCL-20, CCL-21, and IFN-Is (Parmar et al., 2018). Interestingly, a baculovirus-induced innate immunity also provided complete protection against subsequent *P. berghei* sporozoite infection or existing liver stage infection, and neutralization of IFN-α could abolish this effect (Emran et al., 2018). These studies provide mechanistic supports for some earlier observations that interferon inducers have better protection against *P. berghei* sporozoite-induced infection than that by blood stage infection (Jahiel et al., 1968a; Jahiel et al., 1968b; Jahiel et al., 1970). The prevalent pathways of IFN-I induction are through activation of cytosolic receptors that recognize parasite nucleic acids. A significant amount of parasite RNA was detected in non-hepatocytes during liver stage development (Kurup et al., 2019a), suggesting that DCs and other cells may also be sources of IFN-Is. Together, these studies show that during acute liver stage infection, parasite nucleic acids from the rapidly replicating sporozoites can trigger IFN-I responses and production. The IFN-Is then lead to an influx of leukocytes, including NK, NKT and DCs, to the liver and prime the adaptive immunity, especially CD8^+^ T cells, to subsequently eliminate the parasites. Until now, only parasite RNA has been documented to induce IFN-Is in the hepatocytes. In addition, it has been reported that many sporozoites inoculated into the skin of a mouse migrated to the lymph-nodes and were phagocytosed by CD8α^+^ DCs to induce CD8^+^ T cell responses (Radtke et al., 2015), but whether this process also triggers IFN-I responses requires further investigations.

Sterile protection against *Plasmodium* infection can be achieved through exposure to radiation- or genetically-attenuated sporozoites that are able to infect but cannot replicate within the hepatocyte, or through exposure to sporozoites under chloroquine chemoprophylaxis (Mo and McGugan, 2018). It appears that IFN-Is are part of protective immunity during normal or attenuated sporozoite infection; however, the roles of IFN-Is in infections by sporozoites attenuated by various methods has not been well studied. Late liver stage-arresting and replication-competent (LARC) of genetically-attenuated sporozoites have been shown to provide good cross-stage and cross-species protection in mice (Butler et al., 2011; Vaughan et al., 2018). However, a recent study using *P. yoelii* LARC sporozoites showed that the liver stage-engendered IFN-I signaling impaired hepatic CD8^+^ T cell responses, which is critical for liver stage protection. Compared with wildtype (WT) mice, *Ifnar1* ^−/−^ mice exhibited superior protection due to greater CD8^+^ T cell memory and superior CD8^+^ T effector function (Minkah et al., 2019). However, the protective immune mechanisms in the *Ifnar1* ^−/−^ mice after infections with WT and LARC sporozoites can be quite different, and the observations in IFN-1 signaling-deficient mice infected with defective sporozoites may not represent the true protective immunity in normal malaria infections.

## IFN-Is in Blood Stage Infections

Confounding roles of IFN-Is have been reported during bacterial and viral infections (Carrero, 2013). IFN-Is can be protective against bacterial and viral infections by promoting the induction of TNF-α, nitric oxide, and other cytokines. In contrast, IFN-I signaling can also induce suppression of adaptive immune responses during chronic infection with LCMV and acute infections with intracellular bacteria (Carrero, 2013). During malaria blood stage infections, both positive and negative effects of IFN-Is on protection have also been reported in different rodent malaria models and in human infections, which may mirror the observations in acute and chronic infections of bacteria and viruses.

### Malaria Blood Stage Infection Stimulates IFN-Is Responses

A single sporozoite can generate thousands of merozoites that are released to the bloodstream to invade RBCs. In 1968, almost 10 years after the discovery of IFNs, IFNs were reported to be present in the serum of *P. berghei-*infected mice (Huang et al., 1968). However, in a later study, no IFN activity was detected in adult human sera during malaria infection (majority *P. vivax* infection) using virus titration assay (Rytel et al., 1973). Another early study found that acute *P. falciparum* infection induced strong IFN-I responses, especially IFN-α, in children, which was correlated with both parasitemia and NK cell activity (Ojo-Amaize et al., 1981). The differences in these studies could be due to variation in parasite species, infection stage, the time of measurement, as well as host age. Lack of sensitive methods to directly measure IFN-Is at the time of the studies could also be another reason. Later, human *P. falciparum* schizonts and parasite lysate were shown to activate human pDC and murine DCs to produce IFN-Is through the TLR-9 dependent pathway, and elevated serum levels of IFN-Is were detected in *P. falciparum* patients (Pichyangkul et al., 2004). Similarly, *P. berghei* blood stage infection led to IFN-I production by both mouse pDCs and cDCs (deWalick et al., 2007). Blood stage *P. yoelii* infection induced IFN-I responses by cDC *via* the MDA5 pathway (Wu et al., 2014). In a transcriptomic study of infections with multiple *P. yoelii* strains, TLR3/7, TLR9, cGAS, MDA5, and RIG-I were shown to be the upstream regulators significantly activated on day 1 after *P. y. nigeriensis* N67 infection (Xia et al., 2018). pDCs were primed by activated CD169^+^ macrophages upon STING-mediated sensing of parasites in the bone marrow to generate a robust IFN-I responses in *P. y. yoelii* YM blood stage infection (Spaulding et al., 2016). In controlled human malaria infection (CHMI), IFN-Is could be produced throughout the course of infection (Montes de Oca et al., 2016). However, induction of IFN-I production is parasite strain dependent; for example, *P. yoelii nigeriensis* N67 can stimulate an early peak of IFN-Is, whereas mice infected with *P. y. nigeriensis* N67C or *P. y. yoelii* YM produce low levels of IFN-Is (Wu et al., 2014; Wu et al., 2015a; Wu et al., 2020). The parasitemia in mice infected with *P. y. nigeriensis* N67, *P. y. nigeriensis* N67C and *P. y. yoelii* YM strains are similar at 24 h pi (Pattaradilokrat et al., 2014), suggesting that the differences in IFN-I levels are unlikely due to variation in parasitemia. A C741Y amino acid substitution in the protein trafficking domain of the *P. y. nigeriensis* N67 erythrocyte binding-like (PyEBL) protein was shown to affect host immune response, including IFN-I pathways, T cell activation and IgG switching by enhancing phosphatidylserine (PS) exposure on iRBC surface and phagocytosis (Peng et al., 2020). Other genes including those in a locus of parasite chromosome 13 were also significantly linked to expression of many ISGs (Wu et al., 2015a).

### Protective IFN-I Responses Against Parasitemia and Host Mortality

IFN-Is have been shown to be protective against blood stage malaria parasites including suppression of parasitemia and/or improved host survival (**Figure 3B**). Upon activation of IFN-I signaling, ISGs including chemokines/cytokines and other inflammation-inducing mediators are produced to eliminate parasites residing in the host RBCs. Mice deficient of MDA5 or MAVS had compromised ability to control parasitemia day 6 after injection of *P. y. nigeriensis* N67 iRBCs (Wu et al., 2014). A strong IFN-I transcriptional signature was also found in circulating neutrophils from *P. vivax*-infected patients and in *P. chabaudi*-infected mice (Rocha et al., 2015). IFN-I signaling recruited neutrophils that contributed to parasite control but also caused liver damage (Rocha et al., 2015). By blocking STING- and/or MAVS-mediated IFN-I signaling, strong early TLR7-mediated IFN-I responses during *P. y. yoelii* YM blood stage infection helped the host control the parasitemia as well as lower mortality (Yu et al., 2016) (**Figure 3B**). In addition, increased transcription of ISGs in pDCs was reported after *P. chabaudi* blood stage infection or *in vitro* iRBC stimulation, although minimal effects of pDCs or IFN-I signaling on *P. chabaudi* AS infection were observed (Voisine et al., 2010). The dynamics of parasitemia and host survival are different between mice infected with *P. y. nigeriensis* N67 and *P. chabaudi* AS, but these infections shared elevated levels of IFN-Is 24 h pi (300–500 pg/ml) (Kim et al., 2012; Wu et al., 2020) and reduced days 6 and 7 parasitemia (the differences in parasitemia for some *P. chabaudi* AS infections may not be statistically significant) (Voisine et al., 2010; Kim et al., 2012; Wu et al., 2020). It is important to note that different mouse strains were used in the studies (129Sv/Ev, C57BL/6, BALB/c for *P. chabaudi* and C57BL/6 for *P. yoelii* infections). In addition, TLR7 signaling was the major pathway for IFN-I response in *P. chabaudi* AS infection, whereas MDA5 was the major RNA sensor in *P. y. nigeriensis* N67 infections (Baccarella et al., 2013; Wu et al., 2014). The differences in parasitemia control and host survival in mice infected with different strains of *P. chabaudi* and *P. yoelii* could also be partly due to variations in immune mechanisms controlling these infections. In *P. chabaudi* infection, CD4^+^ T cells appear to play a major role in protective immunity, although B cells and antibodies can also contribute to the protection, including B cell regulation of Th cell responses during primary infection (Langhorne et al., 1990; von der Weid et al., 1994; Xu et al., 2000). For some *P. yoelii* infections, the defense mechanism is mostly mediated by humoral factors in the absence of demonstrable cell-mediated immunity (Murphy and Lefford, 1979; Freeman and Parish, 1981). IFN-Is have also been shown to enhance humoral immunity and promote isotype switching by stimulating dendritic cells *in vivo* (Le Bon et al., 2001). A substitution of C741Y in the *P. y. nigeriensis* N67 was indeed linked to increased levels of IFN-α/β, T helper cell differentiation, and antibody isotype switching (Peng et al., 2020). Stimulation of an early IFN-I response may influence the direction of host immune responses such as CD4^+^ T cell activation and later antibody production. Whether and how IFN-Is regulate host immune mechanisms, including T cell activation and antibody production require additional investigation.

Furthermore, continuous injection of purified recombinant human IFN-α suppressed blood stage parasitemia for two *P. yoelii* strains (265 BY and 17XNL) by inhibiting the production of reticulocytes, which were preferentially invaded by these parasite strains, but not for the strain of *P. vinckei petteri* that infects only mature red blood cells (Vigario et al., 2001). Constant human recombinant IFN-α treatment also resulted in enhanced survival, which was associated with an IFN-γ–mediated decrease of parasitemia, expression of intracellular adhesion molecule-1 (ICAM-1 or CD54) in the brain, and CD8^+^ T cells sequestration (Vigario et al., 2007) (**Figure 3B**). ICAM-1 is a cell surface glycoprotein that is typically expressed on endothelial and immune cells; it plays an important role in cell-cell adhesion, extravasation, signal transduction, and inflammation (Bui et al., 2020). Increased expression of ICAM-I in the brain microvasculature has been implicated in the development of cerebral malaria (CM). Absence of ICAM-1 correlates with a decrease of macrophage and iRBC sequestration in the brain and lung capillaries leading to a less severe thrombocytopenia and reduced mortality (Favre et al., 1999). In addition, administration of murine IFN-β after *P. berghei* ANKA infection increased host survival and improved blood–brain barrier function without altering systemic parasitemia. Injection of IFN-β also downregulated CXCL9 and ICAM-1 expression in the brain, reduced serum TNF-α level, and decreased T-cell infiltration in the brain (Morrell et al., 2011). The observations of protection in different parasite models are quite diverse. It is too early to draw a general conclusion on protective mechanisms. More studies on protection mechanisms are required.

### IFN-I Suppression of DC and T Cell Activation

DCs play a central role as a bridge between innate and acquired immunity (Wykes and Good, 2008). IFN-Is were shown to modulate DC function and impair the development of protective IFN-γ producing CD4^+^ T-bet^+^ T cells for parasite control during *P. berghei* ANKA and *P. chabaudi* AS infections (Haque et al., 2011; Haque et al., 2014). CD4^+^ T cells have been shown to produce IFN-γ and macrophage colony-stimulating factor (M-CSF or CSF1) that are important for the activation and expansion of CD169^+^ macrophages to control malaria blood-stage infection (Fontana et al., 2016; Kurup et al., 2019b). IFN-I signaling impaired CD8^−^ cDC function to prime IFN-γ-producing T helper type 1 (Th1) cells for parasite control (Haque et al., 2014), and IFN-I signaling deficiency promoted CD4^+^ T-cell-dependent parasite control thus reducing the onset of severe clinical symptoms and fatal cerebral pathology (Haque et al., 2011) (**Figure 3B**). IFN-Is and IFN-γ were involved in activation-induced cDC death during *P. berghei* ANKA infection (Tamura et al., 2015). In a non-lethal *P. y. yoelii* 17XNL blood stage infection, IFN-Is directly induced T-bet and Blimp-1 expression to promote T regulatory 1 (Tr1) cell responses (Zander et al., 2016). The Tr1 cells then secreted interleukin 10 (IL-10) and IFN-γ to restrict T follicular helper (Tfh) cell accumulation and limit parasite-specific antibody responses. Furthermore, IFN-Is were shown to suppress innate immune cell function and IFN-γ production by parasite-specific CD4^+^ T cells as well as to promote the development of IL-10-producing Tr1 cells (Montes de Oca et al., 2016). On the other hand, IFNAR1-deficiency accelerated humoral immune response and parasite control by boosting the inducible T cell co-stimulator (ICOS) signaling (Sebina et al., 2016). IFNAR1-signalling also impaired germinal center B-cell formation, Ig-class switching, and Tfh cell differentiation thus impeding the resolution of non-lethal blood-stage infection of *P. y. yoelii* 17XNL and *P. chabaudi* AS.

### IFN-Is Promote Inflammation and Host Death

In other studies, IFN-I signaling appears to promote production of inflammatory cytokines/chemokines and pathogenesis of experimental cerebral malaria (ECM). Key mediators of ECM, including *P. berghei* ANKA-induced brain sequestration of CXCR3^+^-activated CD8^+^ T cells, granzyme B, IFN-γ, CXCL9, and CXCL10, were attenuated in IFN-I signaling deficient mice (Palomo et al., 2013). Microglia from mice infected with *P. berghei* ANKA showed signature of IFN-I signaling that was responsible for activation of microglia, production of CXCL9, CXCL10, CCL8 and CCL12 and pathogenesis of ECM (Capuccini et al., 2016). Similarly, mice lacking IFN-I receptor (*Ifnar1* ^−/−^) had a significant decrease in inflammatory response with low levels of IL-6, CCL2, CCL3, CXCL1, CXCL10 and IFN-α and survived *P. y. yoelii* YM infection (Spaulding et al., 2016). IFNAR1 deficiency protected mice from ECM after *P. berghei* ANKA infection, and IFNAR1 signaling unleashed CD8^+^ T cell effector capacity that was crucial for ECM development (Ball et al., 2013). Deficiency of ubiquitin specific peptidase 15 (USP15), an IFN-I positive regulator, also protected mice from ECM and neuroinflammation (Torre et al., 2017). These studies, particularly *P. berghei* ANKA infections, suggest that IFN-I signaling, likely at days 3–6 pi, promotes inflammatory responses and ECM, and that high levels of IFN-γ, CXCL9, CXCL10, and CD8^+^ T cells likely contribute to the host death. On the other hand, the lethal parasite *P. y. yoelii* YM grows quickly in C57BL/6 mice with limited immune response before mouse death with high parasitemia (~90%) on day 7 pi. High IFN-I levels 24 h pi in the *P. y. yoelii* YM-infected *Mavs* ^−/−^ mice also lead to increased level of IFN-γ and host survival, suggesting that increased inflammatory responses help control parasitemia and enhance host survival (Yu et al., 2016). Therefore, protection in terms of host survival depends on parasite/host models. If an infection induces a strong inflammatory response such as *P. berghei* ANKA infection, blockage of IFN-I signaling may reduce inflammation and enhance host survival. For an infection that does not induce a strong inflammatory response, higher levels of IFN-Is and IFN-γ as well as an elevated inflammatory response may instead inhibit parasitemia and enhance survival. Because immune responses are dynamic, measurements of cytokines/chemokines and cell populations will vary at different time points during the course of infection, which can contribute to reports of contradictory results.

## IFN-I Responses in Human Infections

Studying IFN-I responses in human malaria infections have been mainly based on association of disease severity and/or IFN-I levels in the blood with gene expression level and/or genetic polymorphisms in genes playing a role in IFN-I responses. Microarray, RNA sequencing, single-cell sequencing, and genetic association can be important tools for studying IFN-I responses in human malaria infections. However, host immune responses, including IFN-I responses, will be highly variable among patients because the majority of clinical malaria infections in endemic regions carry multiple parasite strains with diverse genetic backgrounds (Conway et al., 1991; O’Brien et al., 2016; Zhu et al., 2019). Other factors such as the time of infection (usually unknown) and host genetic background will greatly influence the level and dynamics of IFN-I responses.

Consistent with observations in some studies using *P. berghei* ANKA infections (Vigario et al., 2001; Vigario et al., 2007), IFN-Is, particularly IFN-α, appear to be protective in human infections of *P. falciparum*. The pre-antimalarial treatment levels of IFN-α were significantly lower in children with severe malaria than those with mild malaria (Luty et al., 2000). Similarly, lower circulating IFN-α was observed in children with severe malaria anemia (SMA) in Kenya, and two polymorphisms in IFN-α promoters [IFN-α2 (A173T) and IFN-α8 (T884A)] leading to reduced IFN-α production were associated with increased susceptibility to SMA and mortality (Kempaiah et al., 2012). Mild *P. falciparum* malaria following an episode of severe malaria was reported to be associated with induction of the IFN-I pathway in Malawian Children (Krupka et al., 2012). Patients with mild *P. falciparum* malaria in Rwanda had upregulated levels of IFN-Is, IFN-γ, complement system components, and nitric oxide (Subramaniam et al., 2015). These observations suggest a protective role of IFN-Is in malaria disease severity.

In contrast, IFN-I responses have also been associated with immune suppression and severe diseases in human malaria infections. Association between IFNAR1 variants and CM in children from Africa was observed; variants with lower IFNAR1 expression were associated with protection, whereas variants with increased IFNAR1 expression were associated with CM (Aucan et al., 2003; Khor et al., 2007; Ball et al., 2013; Feintuch et al., 2018). This is consistent with the observations that blocking IFNAR signaling can protect infected mice from severe disease symptoms. However, the dynamics of IFN-I levels during infection are unknown. Association between IFNAR1 and malaria susceptibility was also observed in Indian populations (Kanchan et al., 2015). But how these IFNAR1 variants regulate IFN-I responses to affect CM development requires further investigation. Dual transcriptome analyses of the host and parasite genes on samples from 46 malaria-infected Gambian children showed that disease severity was associated with increased expression of granulopoiesis and interferon-γ-related genes as well as with inadequate suppression of IFN-I signaling genes (Lee et al., 2018).

## Potential Explanations for the Conflicting Roles of IFN-Is

IFN-I signaling can be protective leading to suppression of parasitemia and better survival or can inhibit DC and T cell activation to dampen adaptive response during malaria parasite infections. The effects or mechanisms of IFN-Is on parasitemia control and disease severity are complex, depending on both species of parasites and their hosts (parasite and host genetics), the timing and levels of IFN-I production, and possibly the subtypes of IFN-Is. Although the mechanisms of IFN-I in regulating host responses to infections of different malaria parasite species are diverse, some major themes can be established (**Figure 3** and **Supplementary Table S1**). IFN-Is are protective if high levels of IFN-Is are produced early (24 h pi) during an infection. *P. berghei* ANKA-infected C57BL/6 mice injected with IFN-α had significantly (*P <*0.05) higher levels of IFN-γ (772 ± 73 pg/ml, day 9 pi) than those receiving diluent (180 ± 14 pg/ml), reduced parasitemia, and better host survival (Vigario et al., 2007). Elevated levels of IFN-Is (300–500 mg/ml IFN-α) can reduce early (day 6) parasitemia as seen in *P. y. nigeriensis* N67 and *P. chabaudi* AS infections (Voisine et al., 2010; Wu et al., 2014; Wu et al., 2020). In another study, the effect of *Ifnar1* ^−/−^ on *P. chabaudi* AS parasitemia may not be significant, but the day 6 parasitemia is higher in the *Ifnar1* ^−/−^ than those of WT mice (Kim et al., 2012). The protection mechanisms for these infections are largely unknown, probably associated with increased IFN-γ expression at later stages of infection. Additionally, the levels of IFN-γ and IL-6 were also higher in mice with elevated early IFN-Is than infections with low IFN-Is, suggesting that IFN-γ, IL-6 and other cytokines may contribute to the reduction of parasitemia and better host survival (Yu et al., 2016; Wu et al., 2020). In another model, *P. y. yoelii* YM infection of STING or MAVS deficient mice produced much higher peaks of IFN-α/β (~2,500 pg/ml IFN-α and ~1,200 pg/ml IFN-β in MAVS knockout mice) than those observed in WT mice infected with *P. y. nigeriensis* N67 infection 24 h pi, which may provide stronger inhibition of parasite growth, leading to higher survival rates (Yu et al., 2016). In all the cases, IFN-I levels were quickly downregulated to background level after 24 h pi. These observations suggest a positive linkage between early peaks of IFN-Is and parasitemia control. It remains to be determined why *P. y. nigeriensis* N67 infection induces an early IFN-I responses, whereas other parasites such as *P. y. nigeriensis* N67C and *P. y. yoelii* YM infections stimulate only low levels of IFN-Is (~60 pg/ml IFN-α and almost no IFN-β in YM infection) in C57BL/6 mice. Additionally, the molecular mechanism of downregulation of the 24 h IFN-I peak is still unknown.

On the other hand, blockage of IFN-I signaling has also been shown to increase DC and T cell activation and inflammation, which can promote survival or mortality depending on parasite species or strains. IFN-Is can cause immunopathology in acute viral infections (Davidson et al., 2014) and mediate immunosuppression during chronic viral infections (Teijaro et al., 2013; Wilson et al., 2013). Therefore, chronically elevated IFN-Is may explain the observations of immune suppression in various reports of malaria infections. In one study showing inhibition of DC and T cell activation by IFN-I signaling, *Ifnar1* ^−/−^ mice or multiple injections of anti-IFNAR antibodies were used (Zander et al., 2016), which is different from infection of wild type mice. In another study, infection of C57BL/6J mice with *P. y. yoelii* YM produced low level of IFN-α (20–70 pg/ml), as reported in other studies (Yu et al., 2016; Wu et al., 2020); however, infection of *Sting* ^Gt/Gt^ mice did not produce high levels of IFN-Is as observed in the study of (Yu et al., 2016). The reason for the discrepancy is unknown. Some possibilities include the uses of different sources of mice and parasite strains. Similarly, low levels of IFN-α/β were also observed in *P. berghei* ANKA-infected WT C57BL/6 mice 24 h pi (Haque et al., 2011; Haque et al., 2014). Again, depletion or blockage of IFN-I signaling resulted in higher levels of IFN-γ, better parasite control and improved host survival (Haque et al., 2011; Haque et al., 2014). Interestingly, higher levels of IFN-α were observed in wild type mice 48 h pi (~70 pg/ml IFN-α vs ~20 pg/ml at 24 h) and day 4 pi (~200 mg/ml IFN-α). Generally, studies suggesting IFN-I suppression of T cell activation or enhancement of inflammatory chemokines/cytokines used *Ifnar1^-/-^* mice or multiple injections of anti-IFNAR antibodies, which are different from infections having a short time increase in IFN-I level. In these models, the absence of IFN-I signaling due to deficiency of IFNAR or antibody blockage may prompt the host to increase IFN-I production through autocrine feedback mechanisms, leading to activation and/or inhibition of alternative immune pathways that would not occur in infections with normal IFN-I signaling. Indeed, it has been shown that production of IFN-α/β early (24 h pi) during LCMV infection of mice was dependent upon the IFN-α/βR and STAT1, and in the absence of IFN-α/βR and the STAT1, an alternative delayed pathway dependent on a functional IFN-γR was activated to produce IFN-α/β at 48 h pi (Malmgaard, 2004). Additionally, the alternative pathway for induction of IFN-α/β exists in IFN-α/βR^−/−^ and STAT1^−/−^ mice but is absent in IFN-γR^−/−^ mice, suggesting that IFN-γ was involved in the alternative pathway. Similar alternative pathways could exist in the *Plasmodium*-infected *Ifnar1* ^−/−^ mice. However, no systematic measurement of IFN-I levels during infection were done in the studies using *Ifnar1* ^−/−^ mice or injections of anti-IFNAR antibodies. If the feedback alternative pathways of IFN-I responses exist in the infected *Ifnar1* ^−/−^ mice, then high IFN-I levels may still play a role in protection through some unknown/alternative mechanisms. Measurements of the dynamic IFN-I levels during malaria infections will be important in order to understand the roles of IFN-Is in malaria infections.

In summary, an early IFN-I response (24 h pi) can be protective through suppression of parasitemia. Disruption of IFN-I signaling through IFNAR deficiency can be beneficial to the host; however, the levels of IFN-Is during the infections are mostly unknown. Whether elevated levels of IFN-Is are protective against severe diseases likely depends on parasite species/strains and host genetic background. It appears that all the protective effects are mediated through adequate levels of IFN-γ, proper activation of immune cells, and production of antibodies. How an early IFN-I response or the absence of IFN-I signaling regulates IFN-γ and antibody responses during malaria parasite infections require additional investigations.

## Regulation of IFN-I Responses During Malaria Plasmodium Infections

IFN-Is regulate many arms of the host immune responses; chronically elevated IFN-Is can suppress adaptive immunity and may also promote autoimmune diseases (Chen et al., 2020). Therefore, the levels of IFN-Is are also closely regulated by various mechanisms such as ubiquitination, phosphorylation and ADP-ribosylation of molecules in the IFN-I response pathways (Takaoka and Yamada, 2019; Zheng and Gao, 2020). Many molecules have been shown to amplify IFN-I signaling, including induction of STAT1 and IRF9 expression by IFN-γ and interleukin-6 (IL-6) (Ivashkiv and Donlin, 2014). There are also proteins such as suppressor of cytokine signaling 1 and 3 (SOCS1 and SOCS3) and ubiquitin carboxy-terminal hydrolase 18 (USP18) or microRNAs (miRNAs) that can suppress IFN-I responses (Yoshimura et al., 2007; Sarasin-Filipowicz et al., 2009). Because IFN-I responses during malaria infections shared the same pathways as those of viral infections, it can be expected that many regulatory mechanisms of IFN-I production in malaria parasite and virus infections are similar. However, malaria parasites are more complex organisms than viruses, and as a result, IFN-I responses and regulation during malaria parasite infections are likely more sophisticated than those of viruses.

All IFN-I response pathways use downstream transcription factors such as IRFs that regulate gene expression of IFNs. Using IRF deficient mice, many of these IRFs have been shown to play important roles in parasitemia control, disease severity, and ECM symptoms (Gun et al., 2014). Three single nucleotide polymorphisms (SNPs) in the IRF1 gene were shown to be correlated with blood parasite levels in malaria patients from west African, and one SNP (rs10065633) was associated with severe disease (Mangano et al., 2008). However, the molecular mechanisms regulating IFN-I responses during human malaria infections remain largely unknown. Recently, several studies using *P. yoelii* parasites begin to shed light on the mechanisms of IFN-I response regulation. A large number of host genes that interact with many genetic loci on the 14 chromosomes of *P. yoelii*, including clusters of genes involved in IFN-I responses, were identified using ts-eQTL (Wu et al., 2015a). Among the genes identified, a set of randomly selected putative ISGs (*Ak3*, *Fosl1*, *Inpp4a*, *Havcr2*, *Fcgr1*, *Bc016423*, *S1pr5*, *Parp14*, *Satb1*, *Selenbp2*, *Helb*, *Helz2*, and *Lrp12*) were found to inhibit luciferease signals driven by IFN-β promoter, suggesting being negative regulators of the IFN-I signaling pathways (**Figure 2**). As mentioned above, *P.y. nigeriensis* N67 infection stimulates peaks of IFN-α/β approximately 24 h pi, and the IFN-I level quickly declines to background level soon after. These negative regulators likely play a role in the decline of the IFN-I levels. Selected genes from this study were further functionally characterized *in vivo* and *in vitro*. For example, CD40 (or TNF receptor superfamily member 5, TNFRSF5), a receptor expressed on the surfaces of many cell types, was shown to enhance STING protein level and STING-mediated IFN-I responses by affecting STING ubiquitination (Yao et al., 2016). On the other hand, the FOS-like antigen 1 (FOSL1), known as a component of FOS transcription factor, was shown to act as a negative regulator of IFN-I responses (Cai et al., 2017). After poly(I:C) or iRBC stimulation, FOSL1 translocated from the nucleus to the cytoplasm, where it interacted with TRAF3 and TRIF to reduce IRF3 phosphorylation and IFN-I production (**Figure 2**). Therefore, FOSL1 acts as a negative regulator of IFN-I response as well as a transcription factor, depending on its cellular location (Cai et al., 2017). Receptor transporter protein 4 gene (*Rtp4*) gene, another gene identified from the same ts-eQTL analysis, was clustered with ISGs such as *Oas2*, *Dhx58*, *Ifit3*, *Usp18*, *Isg15*, and *Ifi35* (Wu et al., 2015a). RTP4 is induced by IFN-Is and binds to the TBK1 complex where it negatively regulates TBK1 signaling by interfering with the expression and phosphorylation of both TBK1 and IRF3 (He et al., 2020). Interestingly, RTP4 may have a specific role in brain pathology because mice deficient of RTP4 have lower West Nile virus titers in the brain and reduced hemorrhages in the cerebellum of *P. berghei* ANKA-infected mice compared with those of WT mice. Using the same ts-eQTL approach but with mRNAs collected 24 h after infection, a gene called *March1*, a member of the membrane-associated ring-CH-type finger 1 family, was also found to be clustered with ISGs (*Oas1d* and *Isg20*) (Wu et al., 2020). MARCH1 was shown to interact with STING, MAVS, and various regulators of the IFN-I pathways to regulate IFN responses likely through protein ubiquitination. *March1* ^−/−^ mice infected with *P. y. nigeriensis* N67 or *P. y. yoelii* YM had significantly reduced serum IFN-I levels day 1 pi, but had increased number of CD86^+^DC and elevated levels of IFN-γ day 4 pi, and survived longer than WT mice. The results suggest that MARCH1 is an important immune regulator affecting IFN-I responses, T cell activation, and adaptive immunity. Studying malaria infections also reveals a cross regulation mechanism of two different IFN-I response pathways (**Figure 2**). It was found that STING- and MAVS-mediated low level IFN-I responses induced the negative regulator SOCS1 that in turn inhibited the more powerful TLR7/MYD88-mediated IFN-I responses in pDCs during *P. y. yoelii* YM blood stage infection (Yu et al., 2016). Additionally, activation of the AIM2, NLRP3 or adaptor caspase-1 inflammsome pathways enhances IL-1β–mediated MYD88-TRAF3-IRF3 signaling and upregulation of SOCS1 that can inhibit MYD88-IRF7 signaling and IFN-I production in pDCs (Yu et al., 2018). These studies identify several regulators and mechanisms of IFN-I responses during malaria infections, which also contributes to our understanding of IFN-I signaling and regulation in general.

Molecules from malaria parasites may also modulate host IFN-I responses, although little is known about the mechanism of immune modulation by the parasites. A genetic locus on chromosome 13 of *P. yoelii* was significantly linked to expression of a large number ISGs, suggesting one or more parasite genes in the lcous may influnce host IFN-I responses (Wu et al., 2015a). Additionally, a C741Y amino acid substitution in the *P. yoelii* erythrocyte binding-like (PyEBL) protein appears to affect host IFN-I responses. The parasite carrying 741Y PyEBL (*P. y. nigeriensis* N67) stimulates higher levels of early IFN-Is than *P. y. nigeriensis* N67C that has 741C PyEBL (Peng et al., 2020; Wu et al., 2020). The substitution also altered host transcritomic responses, including genes in IFN-I responses, T helper cell activation, and IgG isotype switching (Peng et al., 2020). These observations indicate that parasite molecules can also modulate host IFN-I responses. However, parasite molecules that can stimulate and regulate IFN-I responses remain largely unknown.

## Conclusions

Malaria parasite infection stimulates complex and differentially regulated immune responses, dependent on parasite and host species or strains. The parasite PAMPs and DAMPs are recognized by a number of host receptors. Asymptomatic liver stage infection induces the IFN-I responses, which reaches its peak during late liver stage when the parasite undergoes massive replication. The IFN-I signaling recruits and promotes the immune cells to control the liver stage infection. Parasite RNA appears to be the major ligand to elicit IFN-I responses in the hepatocytes, although parasite DNA may also stimulate IFN-I responses at this stage. It would be interesting to investigate whether parasite DNA and other host sensors are involved in the liver stage IFN-I response. After entering the bloodstream, sporozoites are phagocytized by DCs, which may trigger IFN-I responses in these DCs and lead to a positive feedback loop of IFN-I production. IFN-Is have been shown to be protective as well as deleterious in the blood stage parasite infections. Elevated level of IFN-Is or injection of IFN-Is can lead to the clearance of parasites and/or improved host survival rate. Parasite clearance could be mediated by phagocytosis and/or lysis of iRBCs activated by IFN-I signaling. However, deficiency in IFN-I signaling may also enhance DC function, T cell activation, and adaptive immune responses, which may reduce or aggravate disease severity depending on parasite strains and host genetic background. If an infection causes strong inflammatory response with severe disease symptoms (such as *P. y. nigeriensis N67C* and *P. berghei ANKA*), persistent elevated IFN-I levels may help alleviate disease symptoms by inhibiting T cell activation and suppressing inflammatory responses. Additionally, in many cases of rodent malaria infections (such as *P. y. yoelii* YM infection), the IFN-I levels are low, possibly due to mechanisms of immune evasion or inhibition of immune responses. An early strong IFN-I response can help suppress the rapid parasite growth, buying time for the host to develop protective immunity. Therefore, the roles of IFN-Is in malaria protection are dependent on parasite strains, host genetic background, and the level and timing of IFN-I production.

## Author Contributions

XH, LX, KT, JW, and X-ZS wrote the manuscript. JW and X-ZS conceived and designed the paper. JW created **Figure 1**. XH created **Figure 2** and **Figure 3**. All authors contributed to the article and approved the submitted version.

## Funding

This work was supported by the Division of Intramural Research, National Institute of Allergy and Infectious Diseases (NIAID), National Institutes of Health (NIH), USA.

## Conflict of Interest

The authors declare that the research was conducted in the absence of any commercial or financial relationships that could be construed as a potential conflict of interest.
